# Divergent regulation of lncRNA expression by ischemia in adult and aging mice

**DOI:** 10.1007/s11357-021-00460-9

**Published:** 2021-10-26

**Authors:** Tamás Kaucsár, Beáta Róka, Pál Tod, Phuong Thanh Do, Zoltán Hegedűs, Gábor Szénási, Péter Hamar

**Affiliations:** 1 Institute of Translational Medicine, Semmelweis University, Budapest, Hungary; 2 Institute for Translational Medicine, Medical School, University of Pécs, Pécs, Hungary; 3 Institute of Biophysics, Biological Research Centre, Eötvös Loránd Research Network (ELKH), Szeged, Hungary; 4 Department of Biochemistry and Medical Chemistry, Medical School, University of Pécs, Pécs, Hungary

**Keywords:** lncRNA, Acute kidney injury, Ischemia–reperfusion injury

## Abstract

Elderly patients have increased susceptibility to acute kidney injury (AKI). Long noncoding RNAs (lncRNA) are key regulators of cellular processes, and have been implicated in both aging and AKI. Our aim was to study the effects of aging and ischemia–reperfusion injury (IRI) on the renal expression of lncRNAs. Adult and old (10- and 26–30-month-old) C57BL/6 N mice were subjected to unilateral IRI followed by 7 days of reperfusion. Renal expression of 90 lncRNAs and mRNA expression of injury, regeneration, and fibrosis markers was measured by qPCR in the injured and contralateral control kidneys. Tubular injury, regeneration, and fibrosis were assessed by histology. Urinary lipocalin-2 excretion was increased in old mice prior to IRI, but plasma urea was similar. In the control kidneys of old mice tubular cell necrosis and apoptosis, mRNA expression of kidney injury molecule-1, fibronectin-1, p16, and p21 was elevated. IRI increased plasma urea concentration only in old mice, but injury, regeneration, and fibrosis scores and their mRNA markers were similar in both age groups. AK082072 and Y lncRNAs were upregulated, while H19 and RepA transcript were downregulated in the control kidneys of old mice. IRI upregulated Miat, Igf2as, SNHG5, SNHG6, RNCR3, Malat1, Air, Linc1633, and Neat1 v1, while downregulated Linc1242. LncRNAs H19, AK082072, RepA transcript, and Six3os were influenced by both aging and IRI. Our results indicate that both aging and IRI alter renal lncRNA expression suggesting that lncRNAs have a versatile and complex role in aging and kidney injury. An Ingenuity Pathway Analysis highlighted that the most downregulated H19 may be linked to aging/senescence through p53.

**Supplementary Information:**

The online version contains supplementary material available at 10.1007/s11357-021-00460-9.

## Introduction

Acute kidney injury (AKI) has an increasing incidence worldwide, placing a continuously growing burden on the health care systems [1]. The aging of the population is a major cause of the growing number of AKI cases [2]. AKI is associated with elevated short- and long-term mortality and higher risks for developing chronic kidney disease (CKD) leading to end-stage renal disease (ESRD) in all age groups [1]. However, recovery of renal function is significantly worse in elderly patients [3], who require dialysis more often [2].

Old age is one of the main risk factors for AKI [1, 2]. Elderly patients often have many comorbidities and take numerous medications including potentially nephrotoxic ones [2]. Even in the absence of these risk factors, the kidney becomes more susceptible to severe damage with advancing age as kidney function, the renal reserve, and the regenerative capacity decrease during normal aging [2, 4]. Similarly to humans, aged mice and rats are more susceptible to AKI, including renal ischemia and reperfusion injury (IRI) [5–8], the leading cause of AKI [9].

Long noncoding RNAs (lncRNA) are non-protein-coding RNA transcripts of more than 200 nucleotides in length [10]. LncRNAs regulate all kinds of cellular and biological functions, from development, differentiation and proliferation to cell survival and death [11]. The regulatory function of lncRNAs is mediated by diverse mechanisms such as participation in scaffold formation, influencing cellular localization and activity of proteins and regulation of gene expression at all levels (epigenetic, transcriptional, and post-transcriptional) including genomic imprinting, X-inactivation, translation, and stability of mRNAs [10, 11].

Dysregulation of lncRNAs has been demonstrated in many pathological conditions. Likewise, lncRNAs have been implicated in several types of kidney diseases, including AKI and renal fibrosis [12, 13]. Several lncRNAs were shown to modify renal fibrosis and inflammation by regulating the TGF-β/Smad3 pathway [14–18]. LncRNA profiling was only performed in HK-2 and proximal tubular epithelial cells under hypoxic conditions [19, 20], while little is known about the functional involvement of lncRNAs in renal IRI, in vivo.

LncRNAs play important roles in the aging process. LncRNAs regulate cellular senescence, which has a central role in aging [21]. Many cellular processes that trigger either replicative or premature (upon damage) senescence are under lncRNA control, such as telomere shortening, genomic instability, oxidative stress, expression of senescence-regulatory tumor suppressor genes such as cyclin-dependent kinase inhibitor 2A (p16), cyclin-dependent kinase inhibitor 1 (p21) and cyclin-dependent kinase inhibitor 1B (p27), and senescence-associated secretory phenotype [11, 21].

To our knowledge, there is no data on the lncRNA response to renal IRI in old age. Therefore, our aim was to study the effects of both aging and unilateral IRI on the renal expression of lncRNAs.

## Methods

### Animal studies

Male C57BL/6 (Charles River Laboratories, Sulzfeld, Germany) mice were maintained under standardized (light on 08:00–20:00 h; 40–70% relative humidity, 22 ± 1 °C), specified pathogen-free (SPF) conditions, with free access to standard rodent chow (Altromin standard diet, Altromin International, Lage, Germany) and tap water. All procedures were performed in accordance with guidelines set by the National Institutes of Health (USA), the Hungarian law on animal care and protection. All protocols were approved by the Committee on Animal Welfare of Semmelweis University and the Pest County Government Office (registration numbers: XIV-I-001/2103–4/2012 and 22.1/321/3/2011).

### Ischemia–reperfusion surgery

Experiments were performed on 10-month-old (adult) (*n* = 7) and 26–30-month-old (old) mice (*n* = 8). Since aged C57BL/6 mice may develop malignancies, we examined all mice and excluded those with tumors from the study.

All mice were subjected to unilateral renal ischemia–reperfusion injury (IRI) as described previously [22–24]. Briefly, the experiments were carried out using standard operating procedures. The intra-abdominal temperature was maintained using a heating pad (Supertech Ltd., Pécs, Hungary). The animals were anesthetized by an intraperitoneal (ip.) injection of 80 mg/kg of bodyweight ketamine (CP-Pharma Handelsgesellschaft mbH, Burgdorf, Germany) and 4 mg/kg of bodyweight xylazine cocktail (CP-Pharma Handelsgesellschaft mbH, Burgdorf, Germany). After median laparotomy, the left renal pedicle was exposed and the renal artery and vein were clamped for 20 min. The right kidney was left undisturbed and was used for control purposes in all experiments. Postoperative care included morphine hydrochloride (2.5 mg/kg of bodyweight subcutaneous (sc.) injection after the operation) analgesia and ceftriaxone (Rocephin (Roche Hungary Ltd., Budaörs, Hungary), 20 mg/kg of bodyweight sc. once after surgery) to prevent infectious complications.

### Organ harvest

The experiments were terminated 7 days after IRI. Mice were injected with 5000 U/kg of bodyweight heparin i.p. (Ratiopharm GmbH, Ulm, Germany) and 3 min later, they were sacrificed by cervical dislocation. The chest was opened and after cross-section of the vena cava, blood was collected from the thoracic cavity. Blood was washed out of the whole circulation and the parenchymal organs by injecting 10 mL 4 °C saline transcardially. The kidneys were removed and decapsulated. The upper third part of the kidneys was placed in 500 µl TRI Reagent (TR 118, Molecular Research Center, Inc., Cincinnati, OH, USA), was snap frozen in liquid nitrogen, and kept at − 80 °C for RNA isolation. A 1-mm-thick cross-section of the kidney at the hilus level including all layers of cortex and medulla was fixed in 4% buffered formaldehyde. The next day it was dehydrated and embedded in paraffin (FFPE) for histological analysis. The remaining parts of the kidney were cut into pieces, snap frozen in liquid nitrogen, and kept at − 80 °C for molecular biologic analysis.

### Urinary creatinine and Lcn-2 measurements

Spot-urine was collected from mice on days − 1, 1, 3, and 7. Urine samples were centrifuged (3000 g, 20 min, 4 °C) to remove the sediment, and were stored at − 20 °C.

Urine creatinine concentration was assessed with a colorimetric, enzymatic assay (Diagnosticum Ltd. Budapest, Hungary) in 96-well plates (Greiner Bio-One GmbH, Frickenhausen, Germany) according to the manufacturer’s instructions as described previously [25]. Optical density was measured at 555 nm with the SpectraMax 340 Microplate Spectrophotometer (Molecular Devices, Sunnyvale, USA). Concentrations were calculated with SoftMax® Pro Software (Molecular Devices, Sunnyvale, USA).

Lipocalin-2 (Lcn-2, also called neutrophil gelatinase associated lipocalin (NGAL)) has been demonstrated to be a sensitive marker of tubular epithelial damage [25, 26]. The urinary and plasma Lcn-2 concentrations were determined with a mouse Lipocalin-2/NGAL DuoSet ELISA Development kit (R&D Systems, Inc., Minneapolis, UK) as described previously [25]. Samples were measured in duplicates. The optical density was measured with Victor3 1420 Multilabel Counter (PerkinElmer, Waltham, MA, USA) at 450 nm with wavelength correction set to 544 nm. The Lcn-2 concentrations were calculated with WorkOut software (Dazdaq Ltd., Brighton, UK), using a four-parameter logistic curve-fit. Urinary Lcn-2 concentrations were normalized to urinary creatinine concentrations.

### Plasma urea determination

Approximately 100 μl of blood was collected from the tail vein of mice on days − 1, 1, and 3. On day 7, blood was collected as described above. Plasma was separated by centrifugation (6000 g, 2 min) and stored at − 80 °C.

Plasma urea concentrations were measured by a urease and glutamate-dehydrogenase enzymatic assay with colorimetric detection at 340 nm according to the manufacturer’s protocol (Diagnosticum Zrt., Budapest, Hungary) and determined using a standard curve.

### Histology

Renal tubule injury, apoptosis, regeneration, and inflammation in the outer stripe (OS) were evaluated in 4-mm thick hematoxylin–eosin (HE)-stained sections. Renal tubule injury was assessed based on tubule dilation, casts, and signs of necrosis (cell and nuclear swelling, pale cytoplasm, nuclear dissolution). Apoptotic cells were identified as smaller cells with cytoplasmic condensation (hypereosinophilic cytoplasm), and pyknotic and fragmented nuclei [27]. Regeneration score was based on tubular cells with large nuclei and mitotic cells. Inflammation was determined by the degree of infiltration by mononuclear cells. A histological score of 0 to 4 was given blinded to the origin of the tissue as follows: 0: no lesion, 1: minimal or focal changes affecting less than 20% of the OS, 2: mild changes or the extension of the lesion/regeneration to approximately 25% of the OS, 3: moderate changes or the extension of the lesion/regeneration to less than 66% of the OS, 4: severe changes or the extension of the lesion/regeneration to more than 66% of the OS.

The extent of interstitial fibrosis in the OS was investigated on Masson’s trichrome stained sections. A fibrosis score of 0 to 4 was given blinded to the origin of the tissue as follows: 0: no pathologic interstitial fibrosis, interstitial fibrotic (blue fibers) deposition in 1–25% of the field of view (score 1), 26–50% (score 2), 51–75% (score 3), 76–100% (score 4).

### RNA isolation and quantitative real-time PCR measurements of mRNA expression

Total RNA was extracted from the upper third of the kidney with TRI Reagent® (Molecular Research Center, Inc., Cincinnati, OH, USA) according to the protocol provided by the manufacturer (Chomczynski, 1993). The RNA pellet was dissolved in 100 mL RNase-free water. The RNA concentration and purity was inspected with NanoDrop 2000c spectrophotometer (Thermo Fisher Scientific, Waltham, MA, USA). To investigate RNA integrity, samples were electrophoresed on native 1% agarose gel (Invitrogen Ltd., Paisley, UK) and the 28S and 18S ribosomal RNA fraction integrity was verified by gel electrophoresis. The RNA solutions were kept at − 80 °C until further procedures.

Messenger RNA (mRNA) levels were measured by double-stranded DNA dye–based real-time PCR. One microgram of total renal RNA was reverse–transcribed with random hexamer primers by the High-Capacity cDNA Archive Kit (Applied Biosystems) according to the manufacturer’s protocol. The quantitative real-time PCR reaction was performed with SensiFAST™ SYBR® No-ROX Kit (Bioline Reagents Limited, London, UK), according to the manufacturer’s protocol, by the Bio-Rad C1000™ Thermal Cycler with CFX96™ Optics Module real-time PCR system (Bio-Rad Laboratories, Inc., Hercules, CA, USA). Primer annealing was set to 60 °C. Primers (Table 1) were designed by NCBI/Primer-BLAST online software and synthesized by Integrated DNA Technologies (Integrated DNA Technologies, Inc., Coralville, IA, USA). All measurements were done in duplicates. 18S rDNA was used as endogenous reference. The mRNA expressions were calculated with the relative quantification (ΔΔCq) method, and the efficiency of the quantitative PCR reaction was verified with standard curves.

**Table 1:** Sequences of primers used for measuring the expression of target genes by qPCR

Target	Forward primer	Reverse primer
Fn1	CAGACCTACCCAGGCACAAC	CAGCGACCCGTAGAGGTTTT
Kim-1	AAACCAGAGATTCCCACACG	GTCGTGGGTCTTCCTGTAGC
Pcna	GCACGTATATGCCGAGACCTT	ACGTTAGGTGAACAGGCTCAT
p21	CTGTCTTGCACTCTGGTGTCT	CTTGCAGAAGACCAATCTGCG
Igf2	GACACGCTTCAGTTTGTCTGTT	AAGCAGCACTCTTCCACGA
Igf2r	AAGGACAGGCTCGTTCTGAC	AGTTGGACTTGGCAGTGAGT
p16	CGAACTCGAGGAGAGCCATC	TACGTGAACGTTGCCCATCA
p27	TTCGACGCCAGACGTAAACA	TGCGCAATGCTACATCCAATG
18S	CCAGAATGAGGATCCCAGAA	ACCACCTGAAACATGCAACA

Abbreviations: *Fn1*, fibronectin; *Kim-1*, kidney injury molecule-1; *Pcna*, proliferating cell nuclear antigen; *p21 also p21*^*Cip1*^, cyclin-dependent kinase inhibitor 1; *Igf2*, insulin-like growth factor receptor 2; *Igf2r*, insulin-like growth factor-2 receptor; *p16 also p16*^*INK4a*^, cyclin-dependent kinase inhibitor 2A; *p27 also p27Kip1*, cyclin-dependent kinase inhibitor 1B; 18S ribosomal RNA

### LncRNA profiling

The lncRNA profile of 5 control and 5 IRI kidneys from both age groups was analyzed with the mouse LncRNA Profiler qPCR Array Kit (catalog Nr.: RA930A-1, System Biosciences, Palo Alto, CA, USA), following the description of the manufacturer. Briefly, 1.5-µg total RNA per sample was polyA tailed. Next, dT adapters were annealed to the polyA tailed templates and reverse transcribed into cDNA with random primers. Real-time PCR was performed using Maxima™ SYBR Green qPCR Master Mix (Thermo Fisher Scientific). The assay contained primers for 90 different lncRNAs and 5 additional reference genes were tested. The specificity of the reaction was verified with melting curve analysis. The lncRNA expressions were calculated with the relative quantification (ΔΔCq) method.

Data were analyzed using the Ingenuity Pathway Analysis (IPA) software (Qiagen Inc., Hilden, Germany, https://www.qiagenbioinformatics.com/products/ingenuity-pathway-analysis). Nineteen out of the 90 target lncRNAs of the lncRNA Profiler qPCR Array Kit (Supplementary Table 1) were not registered in the IPA knowledgebase. Thus, the following significantly changed lncRNAs could not be included in the IPA functional context mapping studies: Y RNAs, RepA transcript, linc1242, linc1633, and linc-1610-(med) due to lack of identification for corresponding human genes.

### Statistics

Results are presented as mean ± standard error of the mean (SEM) unless otherwise indicated. Logarithmic transformation was performed if normality test indicated inhomogeneity of variances. Continuous variables were analyzed using either one-way or two-way analysis of variance (ANOVA), followed by Tukey’s multiple comparisons test. The null-hypothesis was rejected if the *p* value reached statistical significance (*p < 0.05, **p < 0.01, ***p < 0.001, ****p < 0.0001). LncRNAs were regarded as significantly changed when the age and/or the ischemia effect had a significance level of *p* < 0.05 as determined by two-way ANOVA.

## Results

### Renal parameters altered by aging in the control kidney

**Fig. 1:**
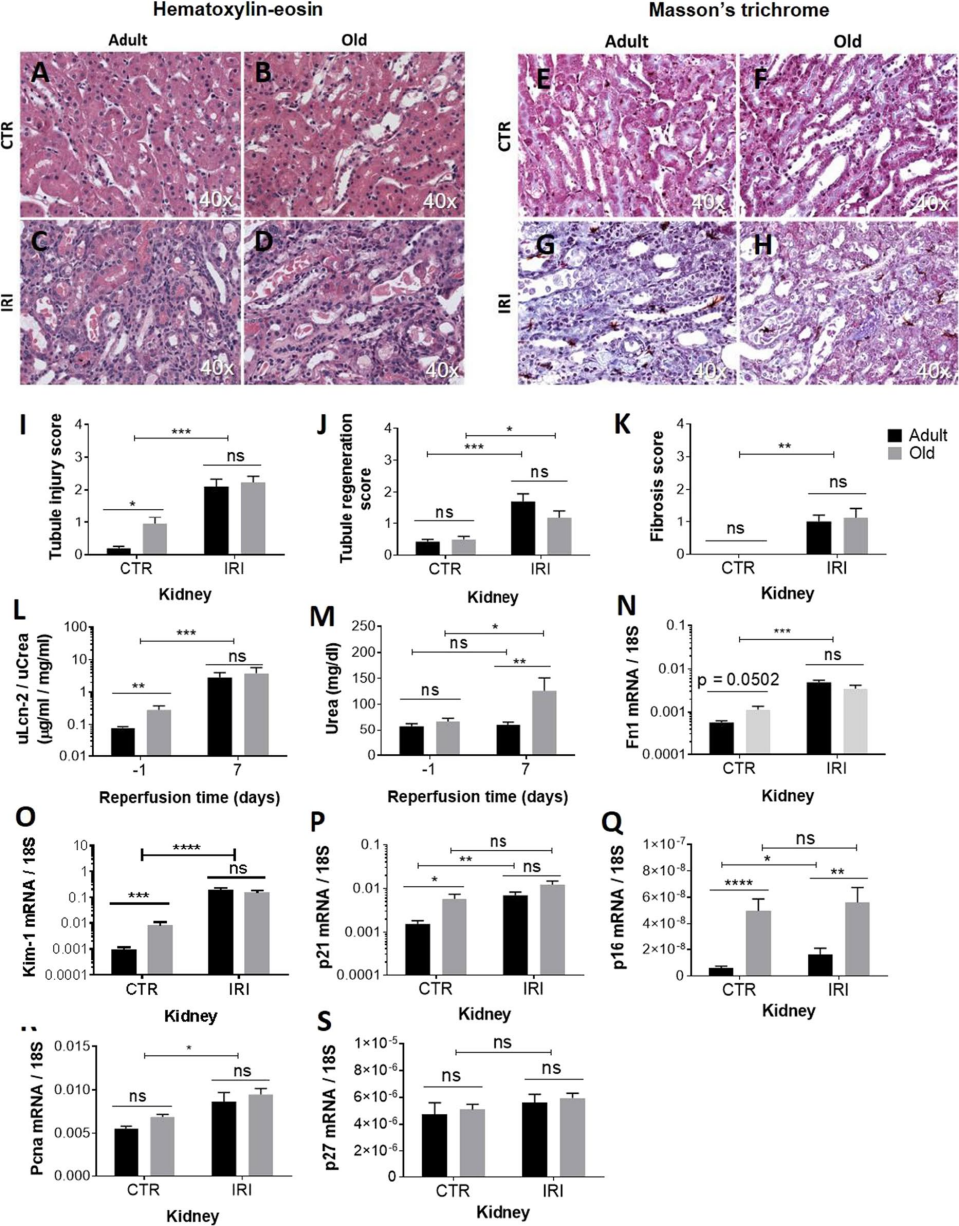
The effects of aging and ischemia on renal morphology and function

Mild morphological deteriorations such as tubule dilation and tubular cell necrosis and apoptosis were detected on HE-stained control kidney slides of old mice (Fig. 1A, B, and I). Tubule regeneration on HE-stained kidney slides was similar in the two age groups (Fig. 1J). There was no visible fibrosis in Masson’s trichrome-stained control kidney slides of old and adult mice (Fig. 1E, F, and K ). The base-line urinary excretion of Lcn-2 was significantly higher in the old group (Fig. 1L); however, base-line plasma urea concentrations were not different in adult and old mice (Fig. 1M). Old age increased the renal mRNA expression of fibronectin (Fn1, Fig. 1N), kidney injury molecule-1 (Kim-1, Fig. 1O), p21 (Fig. 1P), and p16 (Fig. 1Q) but not of proliferating cell nuclear antigen (Pcna, Fig. 1R) and p27 (S).

Adult (age: 10 months) and old (age: 26–30 months) mice subjected to unilateral renal ischemia–reperfusion injury (IRI). Contralateral right kidneys served as controls (CTR). Representative pictures of hematoxylin–eosin (HE) (A–D) and Masson’s trichrome (E–H)-stained kidney Sects. (40 × magnification). Tubular injury (I) and tubule regeneration (J) scores based on the hematoxylin–eosin-stained kidney sections. Fibrosis score (K) based on the Masson’s trichrome-stained sections. Urinary Lcn-2 excretion (L) and plasma urea concentrations (M) 1 day before and 7 days after IRI. Renal Fn1 (N), Kim-1 (O), p21 (P), p16 (Q), Pcna (R), and p27 (S) mRNA expression 7 days after IRI. Two-way ANOVA; ns, not significant; *p < 0.05, **p < 0.01, ***p < 0.001, ****p < 0.0001.

### Renal parameters altered by IRI

Twenty-minute renal ischemia and 7-day reperfusion led to pronounced signs of tubular injury, regeneration, and consequent fibrosis with functional impairment in both age groups. Ischemia induced tubule dilation with casts, firm tubule necrosis, and moderate apoptosis as assessed in HE-stained kidney sections (Fig. 1C,D, and I). Tubule regeneration on HE-stained kidney slides increased after IRI (Fig. 1J). Significant fibrosis was detected on Masson’s trichrome-stained slides following IRI (Fig. 1G, H, and K). There was a strong elevation in urinary Lcn-2 excretion after IRI (Fig. 1L). The mRNA expression of Fn1 (Fig. 1N), Kim-1 (Fig. 1O), p21 (Fig. 1P), and Pcna (Fig. 1R) increased after IRI. All these parameters were similar in adult and old mice. Furthermore, 7 days after ischemia, plasma urea levels were elevated only in the old group, and not in the adult group (Fig. 1M). At the same time, p16 increased only in the adult group, while it was not further elevated in old mice after IRI (Fig. 1Q).

### Renal lncRNA profile of adult and old mice prior to and after renal IRI

**Fig. 2:**
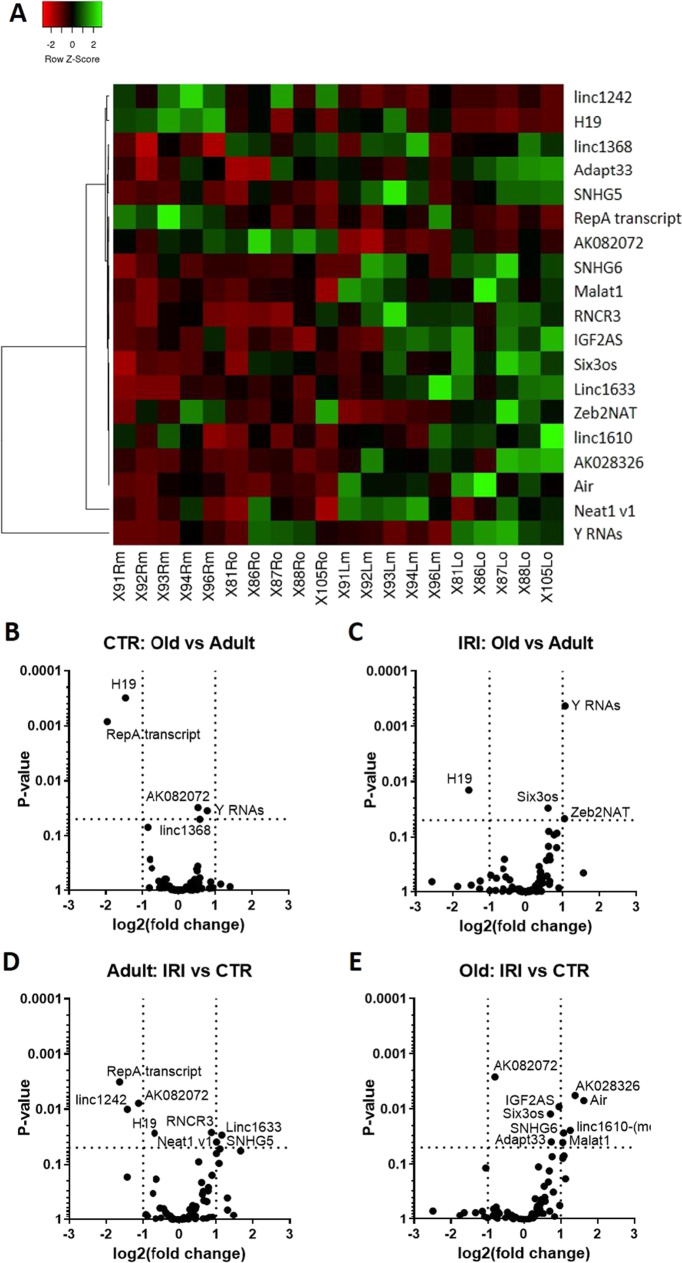
LncRNA profiling in the kidneys. Adult (age: 10 months) and old (age: 26–30 months) mice subjected to unilateral renal ischemia–reperfusion injury (IRI). Contralateral right kidneys served as controls (CTR). (A) Heat map of significantly changed lncRNAs (Rm, right kidney from adult mice; Ro, right kidney from old mice; Lm, left kidney from adult mice; Lo, left kidney from old mice). The effects of age (B, C) in the control (B) and in the IRI (C) kidneys. The effects of IRI (D, E) in the kidneys of adult (D) and of old (E) mice. (B–E): horizontal lines mark *p* = 0.05; vertical lines mark FC = 2x. One-way ANOVA

**Fig. 3:**
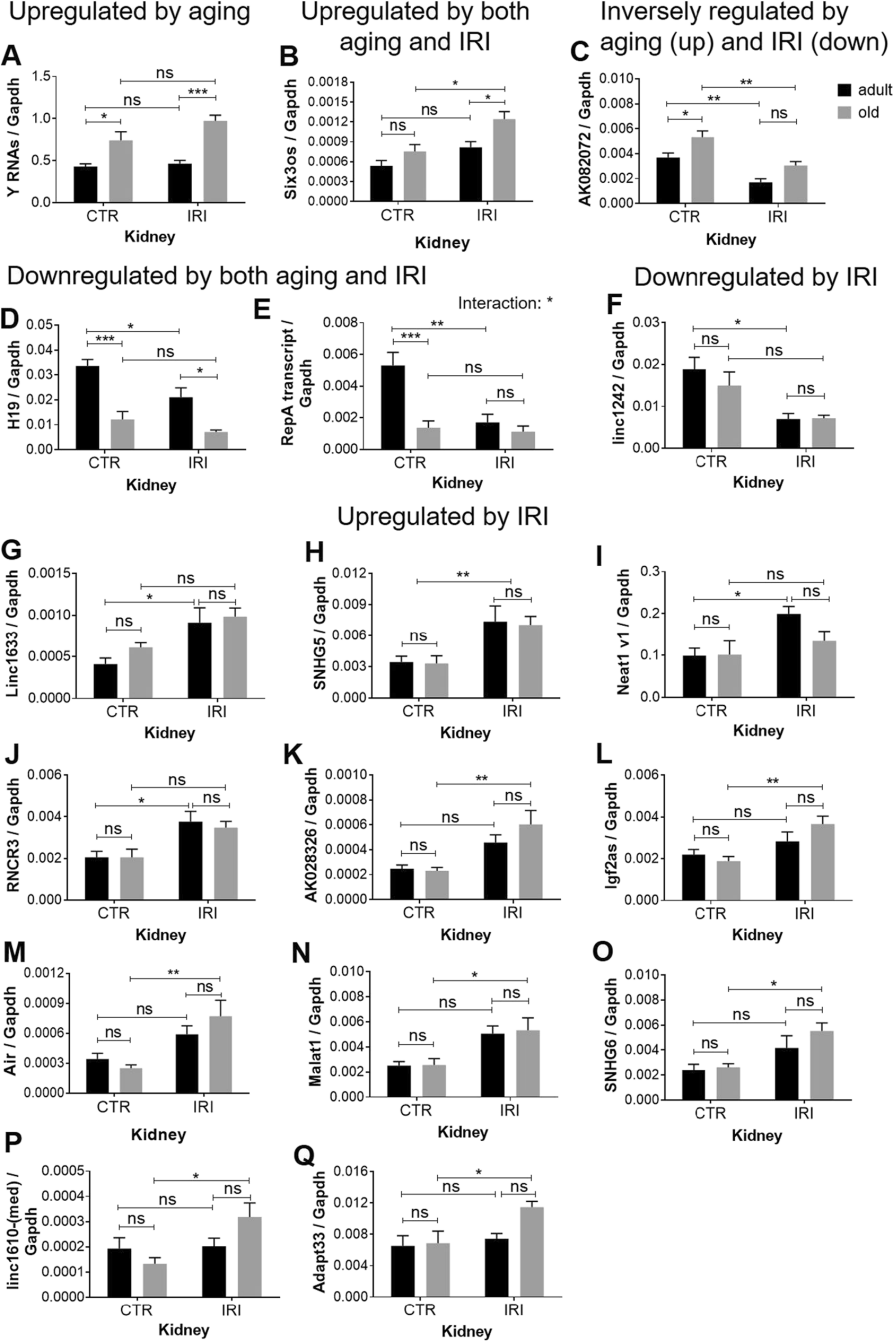
Renal expression of lncRNAs altered by IRI and/or aging. (A) Y RNAs, (B) Six3os, (C) AK082072, (D) H19, (E) RepA transcript, (F) linc1242, (G) Linc1633, (H) SNHG5, (I) Neat1 v1, (J) RNCR3, (K) AK028326 (Miat), (L) Igf2as, (M) Air, (N) Malat1, (O) SNHG6, (P) linc1610-(med), (Q) Adapt33. Adult (age: 10 months) Adapt33 and old (age: 26–30 months) mice subjected to unilateral renal ischemia–reperfusion injury (IRI). Contralateral right kidneys served as controls (CTR). LncRNA expression was normalized to GAPDH. Two-way ANOVA; ns, not significant; *p < 0.05, **p < 0.01, ***p < 0.001 (Tukey’s multiple comparisons test)

The qPCR array included 90 lncRNAs. We excluded 6 lncRNAs due to the lack of amplification, and further 3 lncRNAs because of unspecific reaction products, leaving 81 valid lncRNAs for further analysis. Gapdh varied the least among the reference genes included on the qPCR array; thus, lncRNA expressions were normalized to Gapdh. Figure 2 shows the heatmap of altered lncRNAs and volcano plots of pairwise comparisons. Altogether, 17 lncRNAs changed significantly (two-way ANOVA, *p* < 0.05, Fig. 3, Table 2). In old non-ischemic (CTR) kidneys compared to adult kidneys (Fig. 2B), H19 (Fig. 3D) and RepA transcript (Fig. 3E) were downregulated (FC > 2) and Y RNAs (Fig. 3A) and AK082072 (Fig. 3C) were upregulated significantly but with FC < 2. Furthermore, H19 (down) and Y RNAs (up) were similarly regulated both by age (Fig. 2B) and by IRI (Fig. 2C). On the other hand, in adult mice, IRI led to the downregulation of RepA transcript, Linc1242 (Fig. 3F), and AK082072, and to the upregulation of Linc1633 (Fig. 3G), SNHG5 (Fig. 3H), Neat1 v1 (Fig. 3I) (FC > 2), and RNCR3 (Fig. 3J, FC > 1.5) (Fig. 2D). AK082072 was downregulated by ischemia in both adult (Fig. 2D) and old (Fig. 2E) kidneys. This was the only downregulated lncRNA by ischemia in old mice. However, Six3os (Fig. 3B), AK028326 (Miat) (Fig. 3K), Igf2as (Fig. 3L), Air (Fig. 3M), Malat1 (Fig. 3N), SNHG6 (Fig. 3O), linc1610 (Fig. 3P), and Adapt33 (Fig. 3Q) were all upregulated (FC > 1.5) by IRI in old mice. H19 was downregulated both by age (Fig. 2B) and by IRI in both adult and old mice (Fig. 2C, D), whereas RepA transcript was downregulated both by age (Fig. 2B) and by IRI only in adult mice (Fig. 2D).

**Table 2:** Summary of lncRNA changes

	lncRNA	CTR:old vs CTR:adult	IRI:old vs IRI:adult	IRI:adult vs. CTR:adult	IRI:old vs. CTR:old
1	Y RNAs	Upregulated	Upregulated		
2	Six3os		Upregulated		Upregulated
3	AK082072	Upregulated		Downregulated	Downregulated
4	H19	Downregulated	Downregulated	Downregulated	
5	RepA transcript	Downregulated		Downregulated	
6	Linc1242			Downregulated	
7	Linc1633			Upregulated	
8	SNHG5			Upregulated	
9	Neat1 v1			Upregulated	
10	RNCR3			Upregulated	
11	AK028326 (Miat)				Upregulated
12	Igf2as				Upregulated
13	Air				Upregulated
14	Malat1				Upregulated
15	SNHG6				Upregulated
16	linc1610-(med)				Upregulated
17	Adapt33				Upregulated

### Ingenuity Pathway Analysis

**Fig. 4:**
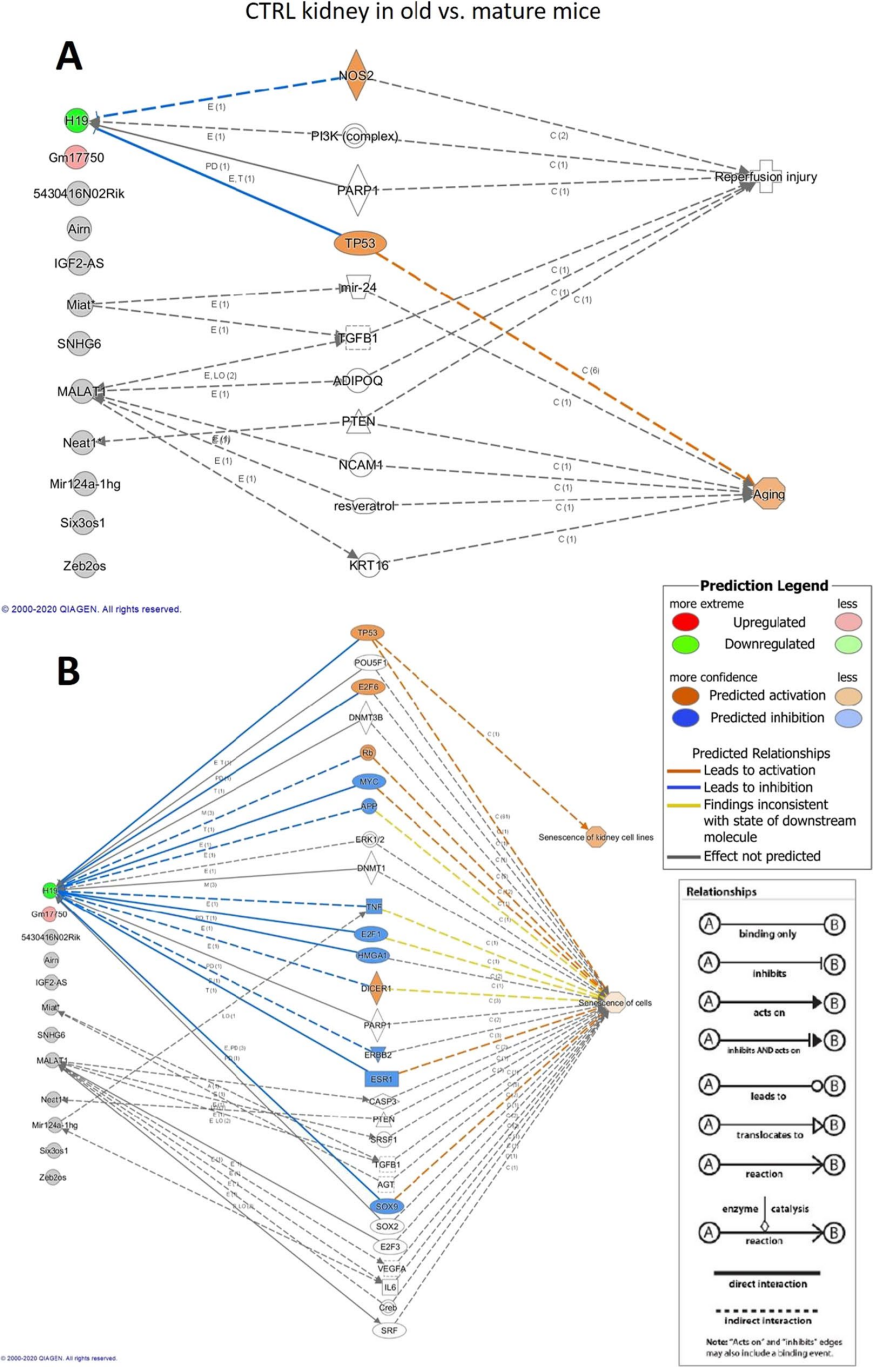
IPA analysis supports the potential functional connection between significantly influenced lncRNAs and aging (A) and senescence (B) in control (non-ischemic) kidneys. miR-124a-1gh: Rncr3, 5430416N02Rik: Adapt33, Gm17750: AK082072

An investigation done by Ingenuity Pathway Analysis (IPA) software demonstrated that H19 downregulation was linked to aging through p53/TP53 (Fig. 4A). IPA associated senescence with H19 through several pathways including p53 in old (non-ischemic) kidneys (Fig. 4B).

**Fig. 5:**
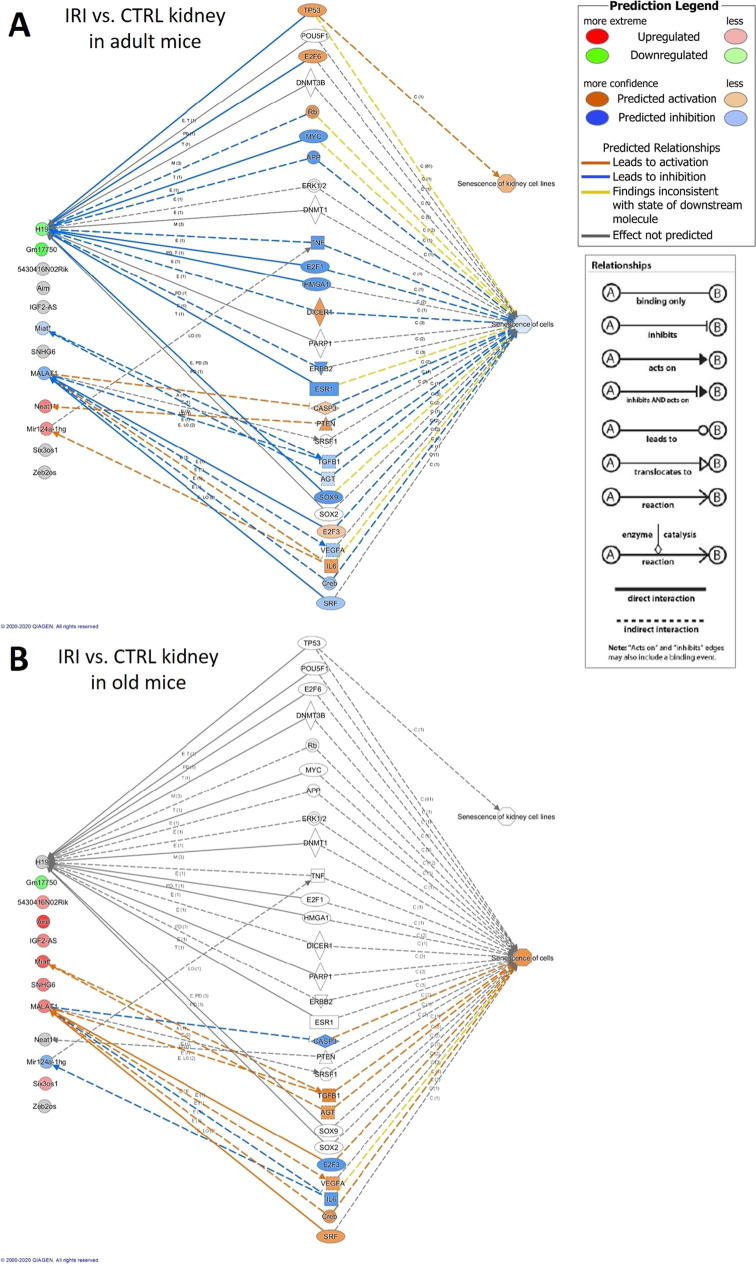
IPA analysis supports the potential functional connection between significantly influenced lncRNAs and senescence in injured vs. control kidneys in adult (A) and old (B) mice. miR-124a-1gh: Rncr3, 5430416N02Rik: Adapt33, Gm17750: AK082072

The IPA analysis predicted a different activation pattern in the network of functional connections between lncRNAs and senescence when adult or old mice were investigated. In adult mice, downregulation of H19 was linked to senescence of kidney cells. IPA predicted that IRI promoted senescence through the upregulation of Miat/AK028326 and Malat1 in old mice (Fig. 5).

## Discussion

In the present study, we detected renal tubular dysfunction in normal kidneys of aged mice based on mild tubular dilation, tubular cell death, upregulation of Kim-1 mRNA, and urinary Lcn-2 excretion. Kim-1 and Lcn-2 are proximal and distal tubular damage markers, respectively. Senescence was also evident in the kidneys of old mice (increased renal p16 and p21 mRNA). Although Fn1 gene expression was elevated too, fibrotic deposition was similar in old and adult mice. Despite the above signs of renal aging, renal function was not reduced in old mice as their baseline plasma urea concentration was not higher than in adult mice. Following unilateral ischemia–reperfusion injury (IRI), plasma urea was not elevated in adult mice as the contralateral kidney compensated for the loss in functional nephrons. However, plasma urea significantly increased in aged mice after IRI, most probably due to lower renal reserve that occurs during aging [2, 4]. Most other functional parameters were similar after IRI in the two age groups. Unexpectedly, the senescence marker p27 mRNA was similar in the kidneys of adult and old mice, and did not change after IRI, although renal p27 protein expression was shown to increase in mice after 7 days of unilateral ureteral occlusion [28] and in diabetes [29]. However, p27 is mainly regulated at the post-transcriptional level through the regulation of its degradation [30].

Ischemia had a more profound effect on expression of lncRNAs than age. We observed the strongest effect in the kidneys of old mice after IRI, as 8 lncRNAs (AK08326, Air, IGF2AS, Six3os, SNHG6, linc1610, Adapt33, and Malat1) were upregulated, while only 4 lncRNAs were upregulated by ischemia in adult mice. The lncRNA profile in postischemic kidneys of old mice is unique, as no previous studies have investigated the complex lncRNA response to renal IRI in old age. H19 and RepA transcripts were downregulated by both age and IRI, whereas, AK082072 was downregulated by IRI in both adult and old mice.

Ingenuity Pathway Analysis (IPA) demonstrated that H19 downregulation was linked to aging through p53/TP53. There is a counterregulatory relationship between p53 and H19, as p53 represses H19 expression and H19 suppresses p53 activation [31]. This inverse regulation was observed in several pathological conditions [32, 33]. Also, p53 is known to be dysregulated during aging [34]. Enhanced p53 activity leads to premature aging in mice [35]. Furthermore, p53 induces cellular senescence by activating p21 [36].

Besides old age, IRI also suppressed H19 and upregulated p16 and p21 expression in adult mice. Suppression of H19 correlated with the upregulation of p16 [37] and p21 [37, 38] in other studies. H19 was found to be upregulated and promote renal fibrosis in mice after unilateral ureteral obstruction [39]. However, H19 seems to be differently regulated by IRI and unilateral ureteral obstruction as it was downregulated in the IRI-injured kidneys.

In accordance with our results, both aging and IRI were accompanied by elevated renal p16 and p21 expression in mice [40, 41]. However, p16 and p21 were significantly upregulated by IRI only in adult mice. Enhanced expression of p21 was found to be protective in the early phases of renal IRI [42, 43]. Also, fibrosis was exacerbated after unilateral ureteral obstruction in p21-deficient mice [44]. Thus, the lack of IRI effect on p21 in old mice might worsen kidney injury.

Besides H19 [37, 38], and SNHG6 [45–47], Miat [48] and Malat1 [49–52] correlated with the regulation of the senescence-regulatory tumor suppressor gene p21. Miat was also associated with p16 [48, 53]. LncRNA SNHG6 was demonstrated to repress p21 [45–47]. Contrary to p21, lncRNA SNHG6 was significantly upregulated by IRI only in old mice. Therefore, we hypothesize that in aged mice, the lack of IRI-induced p21 induction can be related to the upregulated SNHG6 expression. Furthermore, SNHG6 was found to activate the TGF-β/Smad signaling pathway [54, 55] that has a central role in renal fibrogenesis [56]. Although on day 7 there was no difference in the extent of renal fibrosis between the two age groups, upregulation of SNHG6 in the kidneys of old mice may enhance fibrosis on the long term.

AK028326 (also known as RNCR2 or Gomafu), generally called myocardial infarction associated transcript (Miat), was described as a pro-fibrotic and pro-apoptotic lncRNA following myocardial ischemia [57, 58]. Furthermore, Miat was found to upregulate TGF-β expression (59). Miat was also associated with kidney fibrosis [60] and high glucose-induced renal tubular epithelial injury [61]. Here we show for the first time that Miat was upregulated by renal IRI. Obviously, enhanced expression of Miat may contribute to the development and progression of the IRI-induced renal fibrosis.

In accordance with previous findings [23, 62, 63], Malat1 was upregulated after renal IRI, although it had a minor impact on the IRI-induced renal damage [23]. Contrary to our results in the kidney, Malat1 and p21 were found to be inversely regulated in various carcinomas [49–52]. Malat1 is also known to suppress p53 [49], another senescence-regulatory tumor suppressor gene. Further studies are needed to determine how Malat1 and p21 interact in the setting of renal IRI.

Renal expression of RepA was downregulated by both IRI and aging. RepA has a role in X-chromosome inactivation [64]. It is one of the lncRNAs that bind to the histone methyltransferase polycomb repressive complex 2 (PRC2) [65]. Besides X-inactivation, PRC2 also plays a role in the DNA damage response, DNA replication, and the regulation of senescence. Deregulation of PRC2 has been identified in malignancies, and also in aged or stressed cells [66].

Igf2as was upregulated by IRI in the kidneys of old mice. Igf2as inhibition was found to be protective in different disease models by inducing cardiac angiogenesis in type 2 diabetes [67] and neuronal growth in neurotoxicity [68]. Also, Igf2as was downregulated in several types of cancers and suggested to act as a tumor suppressor [69]. Taken together, enhanced Igf2as expression seems to inhibit cell growth. In most cases, it was proposed to act by regulating the expression of insulin-like growth factor 2 (Igf2) [67–69]. It was also reported that Igf2as encoded for a protein [70, 71].

Renal expression of Air was upregulated by IRI. LncRNA Air silences insulin-like growth factor-2 receptor (Igf2r) and organic cation transporters 2 and 3 (Slc22a2/Oct2 and Slc22a3/Oct3) [72]. Air also controls the expression of Slc22a2/Slc22a3 and Igf2r in a different way [72]. Renal expression of Slc22a2 was downregulated by IRI [73]. Furthermore, enhanced expression of Air was correlated with the suppression of Wnt/β-catenin, mTOR, and PI3K signaling [74]. β-catenin, mTOR, and PI3K were all found to be downregulated in the kidney after IRI [75, 76]. Therefore, upregulation of Air fits into the observed gene expression changes caused by renal IRI.

Y RNAs and Zeb2NAT were the only lncRNAs that were affected by aging only. Renal expression of Y RNAs was upregulated in old mice in both kidneys. Y RNAs are highly expressed in the kidneys of mice [77] and were shown to be upregulated in various cancers [78]. Y RNAs have been associated with numerous cellular processes involved in aging (e.g., cell proliferation [78], DNA replication, stress responses, RNA quality control [79], inflammation and apoptosis [80]) but their role in aging has not yet been exploited. Zeb2NAT expression was significantly elevated in old IRI kidneys compared to adult IRI kidney. Knockdown of Zeb2NAT was shown to facilitate reprogramming of aged fibroblasts into pluripotent cells [81].

Neat1 was upregulated by IRI in adult mice. Neat1 was upregulated in ischemia-induced AKI in patients [82]. Neat1 was also induced during the progression of renal fibrosis [83].

Renal expression of AK082072 (also called TMEM161B-AS1) was downregulated by IRI and upregulated by aging. AK082072 was found to be expressed primarily in murine brain tissues [84] but its function is unknown.

IRI upregulated Adapt33 in the kidneys of old mice. Adapt33 was described as a stress-inducible lncRNA that was associated with apoptosis [85].

Six3os (Six3 opposite strand) was also upregulated by IRI in the kidneys of old mice. Six3os is involved in embryonic brain and retinal development [86] and adult neurogenesis [87]. It regulates the expression of Six3 and its target genes [88]. Its role in other organs or in aging has not yet been studied.

In our study, IRI upregulated renal expression of RNCR3 (Retinal non-coding RNA3, also known as LINC00599). To date RNCR3 has been found to play a role in diabetic retinopathy [89], atherosclerosis-related vascular dysfunction [90], glioma [91], and prostate cancer [92].

IRI also upregulated renal expression of SNHG5 (Small Nucleolar RNA Host Gene 5). SNHG5 was shown to act as a sponge of miR-154-5p, which silences Pcna mRNA, thereby enhancing Pcna expression [93]. Accordingly, Pcna mRNA was also elevated after IRI.

From the 17 dysregulated lncRNAs, no information is available for Linc1242 (downregulated by IRI), Linc1633, and linc1610-(med) (upregulated by IRI).

One of the strengths of our study is that we used mice aged between 26 and 30 months, which is rare in kidney studies as the age of old mice is 24 months or even less in most studies. This age corresponds to an approximate human age of 70–80 years [94, 95]. Adult mice (10 months old) served as controls, corresponding to an approximate human age of 30–40 years. It was demonstrated before that age had only minimal impact on the early phases of renal injury after IRI in adult (or middle-aged) mice and rats (up to 12 months of age) [96, 97].

In conclusion, our results indicate that both aging and renal IRI alter the expression of several lncRNAs. As none of the lncRNAs showed an outstanding change, and as the pattern of the altered lncRNAs is quite diversified, it can be hypothesized that lncRNAs have a versatile and complex role in kidney pathophysiology.

## Supplementary Information

Below is the link to the electronic supplementary material.

Supplementary file1 (DOCX 16 KB)
