# Supplementary material for: Divergent regulation of lncRNA expression by ischemia in adult and aging mice
Source: GeroScience. 2021 Oct 26;44(1):429–45. doi: 10.1007/s11357-021-00460-9 (PMC8811094; doi:10.1007/s11357-021-00460-9)
Supplement: Supplementary file 1 — Supplementary file1 (DOCX 16 KB) [file 11357_2021_460_MOESM1_ESM.docx]

Article title: Divergent regulation of lncRNA expression by ischemia in adult and aging mice Journal name: GeroScience

Author names: Tamás Kaucsár, Beáta Róka, Pál Tod, Phuong Thanh Do, Zoltán Hegedűs, Gábor Szénási, Péter Hamar

Corresponding author: Péter Hamar

Affiliation: Institute of Translational Medicine, Semmelweis University, Budapest, Hungary and Institute for Translational Medicine, Medical School, University of Pécs, Pécs, Hungary E-mail address: [hamar.peter@med.semmelweis-univ.hu](mailto:hamar.peter@med.semmelweis-univ.hu)

Supplementary material Supplementary Table 1.

list of the 90 lncRNAs and the 5 reference genes (grey background) included in the lncRNA Profiler qPCR Array Kit and the 19 lncRNAs not registered in the IPA knowledgebase (strike-through letters, light grey background).

|  | 1 | 2 | 3 | 4 | 5 | 6 | 7 | 8 | 9 | 10 | 11 | 12 |
| --- | --- | --- | --- | --- | --- | --- | --- | --- | --- | --- | --- | --- |
| A | Adapt 33 | Air | AK007 836 | AK141 205 | AK028 326 | AK082 072 | ATIA | antiPe g11 | B2 SINE RNA | BACE1 AS | BC1 | ~~BGn-As~~ |
| B | BORG | CDR1  antise nse | Dio3o s | Dlx1as | Emx2 os | Evf2 | ~~Foxn~~ ~~2-as~~ | GAS5 | Gomafu | Gtl2-as | H19 | ~~H19~~  ~~antisens~~ ~~e~~ |
| C | HOTA IR | HOTTI P | Hoxa1 1as | IGF2A S | Jpx | Kcnq1 ot1 | ~~Linc1~~ ~~242~~ | ~~Linc13~~ ~~31~~ | ~~Linc136~~ ~~8~~ | ~~Linc161~~ ~~2~~ | Linc154 7 | Linc158 2 |
| D | ~~Linc1~~ ~~609-~~  ~~long~~ | ~~Linc1~~ ~~609-~~  ~~short~~ | ~~Linc16~~ ~~10-~~  ~~long~~ | ~~Linc16~~ ~~10-~~  ~~mediu~~ ~~m~~ | ~~Linc16~~ ~~10-~~  ~~short~~ | ~~Linc~~ ~~1623~~ | ~~Linc1~~ ~~633~~ | LincE NC1 | LincRNA Cox2 | LincRN Ap21 | LincRNA Sox2 | LINC - MD1 |
| E | ~~LXRBS~~ ~~V~~ | Malat 1 | mascR NA | MEG3 | MEG9 | ~~MSUR~~ ~~1~~ | Msx1 as | Neat1 v1/ MEN | Neat1 v2/Men b | Nespas | Nkx2.2A S | NRON |
| F | Otx2o s | PINC | PINC  1Kb  iso | Pldi | Reco  m. hot  spot | ~~RepA~~ ~~transc~~  ~~ript~~ | Rian | Rmst | RNCR3 | SCA8 (KLHL1-  AS) | Six3os | Six3oscl one9 |
| G | SNHG 1 | SNHG 3 | SNHG 4 | SNHG 5 | SNHG 6 | Sox2o t | SRA | Tsix | TUG1 | Vax2os 1 | ~~VL30~~ ~~RNAs~~ | WT1-AS |
| H | Xist | ~~Y~~ ~~RNAs~~ | Zeb2N AT | Zfas1 | Zfhx2 as | Mistra l | **18S rRNA** | **RNU43**  **(snoR**  **NA)** | **GAPDH** | **Beta Actin** | **U6**  **snRNA** | No assay control |
